# Poly[[aqua­(μ_4_-1*H*-benzimidazole-5,6-dicarboxyl­ato-κ^4^ *N* ^3^:*O* ^5^:*O* ^5′^:*O* ^6^)(*N*,*N*-dimethyl­formamide-κ*O*)cadmium(II)] dihydrate]

**DOI:** 10.1107/S1600536810003065

**Published:** 2010-01-30

**Authors:** Hao Wang, Shi-Jie Li, Wen-Dong Song, Xiao-Fei Li, Dong-Liang Miao

**Affiliations:** a College of Food Science and Technology, Guang Dong Ocean University, Zhanjiang 524088, People’s Republic of China; b College of Science, Guang Dong Ocean University, Zhanjiang 524088, People’s Republic of China; c College of Agriculture, Guang Dong Ocean University, Zhanjiang 524088, People’s Republic of China

## Abstract

In the title compound, {[Cd(C_9_H_4_N_2_O_4_)(C_3_H_7_NO)(H_2_O)]·2H_2_O}_*n*_, the Cd^II^ atom is coordinated by one N atom and three O atoms from four different 1*H*-benzimidazole-5,6-dicarboxyl­ate (Hbidc) ligands, one O atom from one dimethyl­formamide ligand, and one O atom from a water mol­ecule in a distorted octa­hedral geometry. The Hbidc ligands connect the Cd atoms into a two-dimensional network parallel to (001). N—H⋯O and O—H⋯O hydrogen bonds involving the water molecules are observed in the crystal structure.

## Related literature

For related structures of 1*H*-benzimidazole-5,6-dicarboxyl­ate complexes, see: Song, Wang, Hu *et al.* (2009 ▶); Song, Wang, Li *et al.* (2009 ▶); Song, Wang, Qin *et al.* (2009 ▶); Wang *et al.* (2009 ▶).
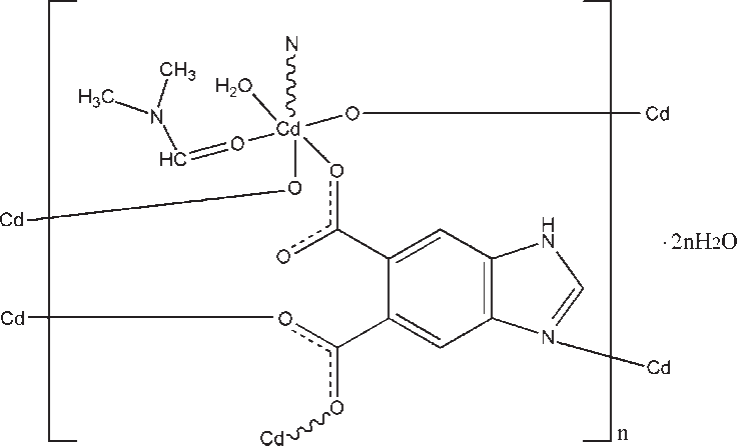


## Experimental

### 

#### Crystal data

[Cd(C_9_H_4_N_2_O_4_)(C_3_H_7_NO)(H_2_O)]·2H_2_O
*M* *_r_* = 443.69
Triclinic, 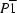

*a* = 7.7729 (16) Å
*b* = 9.1648 (18) Å
*c* = 11.458 (2) Å
α = 102.76 (3)°
β = 97.70 (3)°
γ = 94.96 (3)°
*V* = 783.2 (3) Å^3^
*Z* = 2
Mo *K*α radiation
μ = 1.44 mm^−1^
*T* = 293 K
0.29 × 0.25 × 0.21 mm

#### Data collection

Rigaku/MSC Mercury CCD diffractometer
Absorption correction: multi-scan (REQAB; Jacobson, 1998 ▶) *T*_min_ = 0.680, *T*_max_ = 0.752
6197 measured reflections
2800 independent reflections
1539 reflections with *I* > 2σ(*I*)
*R*_int_ = 0.121

#### Refinement

*R*[*F* ^2^ > 2σ(*F* ^2^)] = 0.086
*wR*(*F* ^2^) = 0.223
*S* = 1.14
2800 reflections
219 parameters
9 restraints
H-atom parameters constrained
Δρ_max_ = 2.12 e Å^−3^
Δρ_min_ = −1.80 e Å^−3^

### 

Data collection: *CrystalStructure* (Rigaku/MSC, 2002 ▶); cell refinement: *CrystalStructure*; data reduction: *CrystalStructure*; program(s) used to solve structure: *SHELXS97* (Sheldrick, 2008 ▶); program(s) used to refine structure: *SHELXL97* (Sheldrick, 2008 ▶); molecular graphics: *ORTEPII* (Johnson, 1976 ▶); software used to prepare material for publication: *SHELXL97*.

## Supplementary Material

Crystal structure: contains datablocks I, global. DOI: 10.1107/S1600536810003065/hy2275sup1.cif

Structure factors: contains datablocks I. DOI: 10.1107/S1600536810003065/hy2275Isup2.hkl

Additional supplementary materials:  crystallographic information; 3D view; checkCIF report

## Figures and Tables

**Table 1:** Hydrogen-bond geometry (Å, °)

*D*—H⋯*A*	*D*—H	H⋯*A*	*D*⋯*A*	*D*—H⋯*A*
O1*W*—H1*W*⋯O2^i^	0.84	1.92	2.757 (11)	177
O1*W*—H2*W*⋯O4^ii^	0.84	1.85	2.649 (12)	159
O2*W*—H3*W*⋯O1*W*	0.84	2.16	2.888 (9)	145
O2*W*—H4*W*⋯O1	0.84	2.00	2.811 (11)	162
O3*W*—H5*W*⋯O2^i^	0.84	2.11	2.810 (12)	140
O3*W*—H6*W*⋯O2*W*^iii^	0.84	2.29	2.766 (14)	117
N2—H2⋯O2*W*^iv^	0.86	2.18	2.970 (16)	152

Symmetry codes: (i) 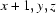; (ii) 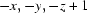; (iii) 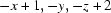; (iv) 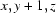.

**Fig. 1.**
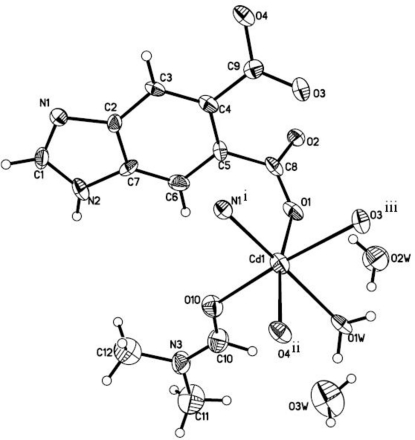
The asymmetric unit of the title compound, showing the 30% probability displacement ellipsoids. [Symmetry codes: (i) -x, 1-y, 1-z; (ii) 1+x, y, z; (iii) -x, -y,1-z.]

**Fig. 2.**
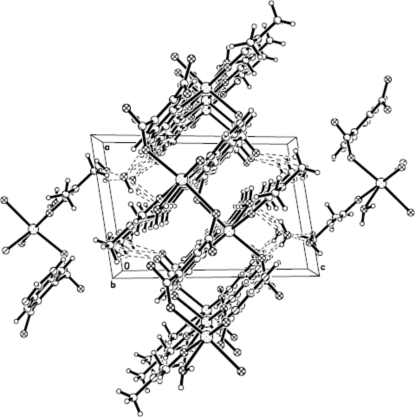
A view of the crystal packing. Hydrogen bonds are shown as dashed lines.

## supplementary crystallographic information

### Comment

From the structural point of view, 1*H*-benzimidazole-5,6-dicarboxylic acid (H_3_bidc) possesses two N atoms of imidazole ring and four O atoms of carboxylate groups and might be used as versatile linker in constructing coordination polymers with abundant hydrogen bonds. Based on this idea, a series of coordination polymers fomed by this ligand have been reported by us: *catena*-poly[[diaqua(1,10-phenanthroline-κ^2^N,N')nickel(II)]- µ-Hbidc-κ^2^N^3^:O^6^] (Song, Wang, Hu *et al.*, 2009), pentaaqua(Hbidc-κN^3^)cobalt(II) pentahydrate (Song, Wang, Li *et al.*, 2009), pentaaqua(Hbidc-κN^3^)nickel(II) pentahydrate (Song, Wang, Qin *et al.*, 2009), and tetraaquabis(Hbidc-κN^3^)cobalt(II) dimethylformamide disolvate dihydrate (Wang *et al.*, 2009). In the present paper, we report the title complex.

As shown in Fig. 1, the Cd^II^ atom exhibits an octahedral coordination geometry, defined by three O atoms from three different Hbidc ligands, one N atom from another Hbidc ligand, one O atom from a dimethylformamide ligand and one O atom from a water molecule. The equatorial plane is defined by O1W, O10, N1^i^ and O3^iii^ atoms, while O1 and O4^ii^ occupy the axial positions [symmetry codes: (i) -*x*, 1-*y*, 1-*z*; (ii) 1+*x*, *y*, *z*; (iii) -*x*, -*y*, 1-*z*]. Two solvent water molecules are present in the asymmetic unit. O—H···O and N—H···O hydrogen bonds are observed in the crystal structure with hydrogen-bond geometry in the normal range (Fig. 2 and Table 1).

### Experimental

A dimethylformamide solution (20 ml) containing CdCl_2_(0.1 mmol) and H_3_bidc (0.2 mmol) was stirred for a few minutes in air, and then left to stand at room temperature. Colorless crystals were obtained in a few weeks.

### Refinement

C- and N-bound H atoms were placed at calculated positions and treated as riding on the parent atoms, with C—H = 0.93 (CH), 0.96 (CH_3_), N—H = 0.86 Å and with *U*_iso_(H) = 1.2(1.5 for methyl)*U*_eq_(C, N). The water H-atoms were located in a difference Fourier map and refined as riding, with a distance restraint of O—H = 0.84 Å and with *U*_iso_(H) = 1.5*U*_eq_(O). The highest residual electron density was found 1.07 Å from Cd1 and the deepest hole 0.97 Å from Cd1.

### Crystal data

**Table:**

[Cd(C_9_H_4_N_2_O_4_)(C_3_H_7_NO)(H_2_O)]·2H_2_O	*Z* = 2
*M**_r_* = 443.69	*F*(000) = 444
Triclinic, *P*1	*D*_x_ = 1.881 Mg m^−^^3^
Hall symbol: -P 1	Mo *K*α radiation, λ = 0.71073 Å
*a* = 7.7729 (16) Å	Cell parameters from 3441 reflections
*b* = 9.1648 (18) Å	θ = 3.3–27.4°
*c* = 11.458 (2) Å	µ = 1.44 mm^−^^1^
α = 102.76 (3)°	*T* = 293 K
β = 97.70 (3)°	Block, colorless
γ = 94.96 (3)°	0.29 × 0.25 × 0.21 mm
*V* = 783.2 (3) Å^3^	

### Data collection

**Table:**

Rigaku/MSC Mercury CCD diffractometer	2800 independent reflections
Radiation source: fine-focus sealed tube	1539 reflections with *I* > 2σ(*I*)
graphite	*R*_int_ = 0.121
ω scans	θ_max_ = 25.2°, θ_min_ = 3.3°
Absorption correction: multi-scan (REQAB; Jacobson, 1998)	*h* = −9→7
*T*_min_ = 0.680, *T*_max_ = 0.752	*k* = −10→10
6197 measured reflections	*l* = −13→13

### Refinement

**Table:**

Refinement on *F*^2^	Primary atom site location: structure-invariant direct methods
Least-squares matrix: full	Secondary atom site location: difference Fourier map
*R*[*F*^2^ > 2σ(*F*^2^)] = 0.086	Hydrogen site location: inferred from neighbouring sites
*wR*(*F*^2^) = 0.223	H-atom parameters constrained
*S* = 1.14	*w* = 1/[σ^2^(*F*_o_^2^) + (0.0683*P*)^2^ + 2.7388*P*] where *P* = (*F*_o_^2^ + 2*F*_c_^2^)/3
2800 reflections	(Δ/σ)_max_ < 0.001
219 parameters	Δρ_max_ = 2.12 e Å^−^^3^
9 restraints	Δρ_min_ = −1.80 e Å^−^^3^

### Fractional atomic coordinates and isotropic or equivalent isotropic displacement parameters (Å^2^)

**Table:**

	*x*	*y*	*z*	*U*_iso_*/*U*_eq_	
Cd1	0.32811 (14)	0.19007 (13)	0.58926 (9)	0.0427 (4)	
O1	0.1397 (12)	0.1778 (11)	0.7248 (7)	0.043 (2)	
O2	−0.1244 (11)	0.1608 (11)	0.7803 (8)	0.043 (2)	
O1W	0.5202 (9)	0.0812 (8)	0.7112 (7)	0.045 (3)	
O10	0.4501 (13)	0.4197 (11)	0.7028 (8)	0.048 (3)	
N1	−0.1716 (14)	0.7274 (13)	0.5549 (9)	0.037 (3)	
N2	0.0418 (16)	0.7573 (12)	0.7100 (10)	0.046 (3)	
H2	0.1247	0.8019	0.7676	0.055*	
N3	0.6296 (17)	0.5826 (15)	0.8605 (11)	0.057 (4)	
C1	−0.0542 (18)	0.8220 (17)	0.6334 (13)	0.044 (4)	
H1	−0.0382	0.9245	0.6362	0.053*	
C2	−0.1506 (16)	0.5900 (15)	0.5837 (12)	0.035 (3)	
C3	−0.2450 (15)	0.4482 (15)	0.5278 (10)	0.031 (3)	
H3	−0.3353	0.4370	0.4633	0.037*	
C4	−0.1988 (17)	0.3261 (16)	0.5722 (11)	0.037 (3)	
C5	−0.0634 (17)	0.3421 (14)	0.6700 (11)	0.033 (3)	
C6	0.0295 (17)	0.4860 (17)	0.7241 (12)	0.045 (4)	
H6	0.1203	0.4999	0.7887	0.054*	
C7	−0.0192 (16)	0.6032 (16)	0.6778 (10)	0.033 (3)	
C8	−0.0144 (18)	0.2164 (15)	0.7258 (12)	0.039 (3)	
C11	0.758 (3)	0.606 (2)	0.9664 (14)	0.086 (6)	
H11A	0.7649	0.5123	0.9907	0.128*	
H11B	0.7250	0.6793	1.0306	0.128*	
H11C	0.8693	0.6407	0.9491	0.128*	
C12	0.587 (2)	0.713 (2)	0.8183 (16)	0.077 (6)	
H12A	0.5613	0.6868	0.7313	0.116*	
H12B	0.6835	0.7907	0.8445	0.116*	
H12C	0.4859	0.7477	0.8506	0.116*	
C10	0.560 (2)	0.4477 (19)	0.7957 (13)	0.054 (4)	
H10	0.5975	0.3655	0.8226	0.065*	
O3W	0.7681 (13)	0.2252 (9)	1.0089 (11)	0.127 (6)	
H5W	0.8465	0.2124	0.9650	0.190*	
H6W	0.6998	0.1446	0.9891	0.190*	
O2W	0.2935 (7)	0.0032 (12)	0.8739 (9)	0.080 (4)	
H3W	0.3906	0.0207	0.8522	0.120*	
H4W	0.2273	0.0510	0.8364	0.120*	
C9	−0.2912 (17)	0.1756 (17)	0.5120 (11)	0.036 (3)	
O3	−0.2155 (12)	0.0562 (10)	0.5048 (7)	0.041 (2)	
O4	−0.4532 (9)	0.1698 (8)	0.4625 (7)	0.043 (2)	
H1W	0.6279	0.1081	0.7315	0.065*	
H2W	0.4982	−0.0084	0.6711	0.065*	

### Atomic displacement parameters (Å^2^)

**Table:**

	*U*^11^	*U*^22^	*U*^33^	*U*^12^	*U*^13^	*U*^23^
Cd1	0.0451 (7)	0.0367 (7)	0.0457 (6)	−0.0036 (5)	0.0070 (4)	0.0115 (5)
O1	0.040 (6)	0.042 (6)	0.043 (5)	−0.015 (5)	0.005 (4)	0.013 (4)
O2	0.033 (5)	0.041 (6)	0.059 (6)	−0.002 (5)	0.007 (4)	0.024 (5)
O1W	0.040 (5)	0.038 (6)	0.049 (5)	−0.017 (5)	0.006 (4)	0.004 (5)
O10	0.054 (6)	0.044 (7)	0.039 (5)	−0.003 (5)	−0.005 (5)	0.006 (5)
N1	0.035 (6)	0.030 (7)	0.042 (6)	0.000 (6)	0.000 (5)	0.006 (5)
N2	0.056 (8)	0.023 (7)	0.053 (7)	−0.007 (6)	0.011 (6)	0.002 (6)
N3	0.062 (9)	0.034 (8)	0.064 (8)	0.003 (7)	−0.009 (7)	0.004 (7)
C1	0.048 (9)	0.035 (9)	0.064 (9)	0.014 (7)	0.021 (8)	0.031 (8)
C2	0.030 (7)	0.029 (8)	0.057 (8)	0.011 (6)	0.020 (6)	0.020 (7)
C3	0.025 (6)	0.043 (9)	0.023 (6)	−0.010 (6)	0.001 (5)	0.010 (6)
C4	0.035 (8)	0.040 (9)	0.034 (7)	−0.009 (7)	0.003 (6)	0.009 (6)
C5	0.045 (8)	0.022 (7)	0.033 (7)	0.002 (6)	0.006 (6)	0.008 (6)
C6	0.032 (8)	0.055 (10)	0.043 (8)	−0.006 (7)	−0.007 (6)	0.016 (7)
C7	0.029 (7)	0.047 (9)	0.029 (7)	0.016 (7)	0.003 (5)	0.016 (6)
C8	0.041 (9)	0.027 (8)	0.043 (8)	−0.011 (7)	0.010 (6)	−0.002 (6)
C11	0.105 (16)	0.080 (15)	0.052 (10)	0.008 (12)	−0.028 (10)	0.001 (10)
C12	0.083 (14)	0.063 (14)	0.078 (12)	0.011 (11)	0.036 (10)	−0.013 (10)
C10	0.065 (11)	0.049 (11)	0.049 (9)	0.012 (9)	0.009 (8)	0.009 (8)
O3W	0.171 (17)	0.126 (15)	0.085 (10)	−0.006 (12)	0.049 (10)	0.020 (10)
O2W	0.075 (8)	0.090 (10)	0.088 (8)	0.009 (7)	0.027 (7)	0.041 (8)
C9	0.037 (8)	0.044 (9)	0.030 (7)	0.006 (7)	0.008 (6)	0.015 (6)
O3	0.056 (6)	0.028 (6)	0.039 (5)	0.000 (5)	0.011 (4)	0.005 (4)
O4	0.044 (6)	0.037 (6)	0.041 (5)	−0.009 (5)	0.008 (4)	−0.001 (4)

### Geometric parameters (Å, °)

**Table:**

Cd1—N1^i^	2.226 (11)	C3—C4	1.382 (19)
Cd1—O10	2.266 (9)	C3—H3	0.9300
Cd1—O1	2.287 (8)	C4—C5	1.404 (18)
Cd1—O3^ii^	2.314 (9)	C4—C9	1.472 (18)
Cd1—O1W	2.344 (9)	C5—C6	1.414 (18)
Cd1—O4^iii^	2.373 (7)	C5—C8	1.489 (19)
O1—C8	1.278 (16)	C6—C7	1.359 (19)
O2—C8	1.261 (14)	C6—H6	0.9300
O1W—H1W	0.8380	C11—H11A	0.9600
O1W—H2W	0.8382	C11—H11B	0.9600
O10—C10	1.236 (17)	C11—H11C	0.9600
N1—C1	1.301 (17)	C12—H12A	0.9600
N1—C2	1.388 (17)	C12—H12B	0.9600
N2—C1	1.346 (17)	C12—H12C	0.9600
N2—C7	1.400 (17)	C10—H10	0.9300
N2—H2	0.8600	O3W—H5W	0.8411
N3—C10	1.320 (19)	O3W—H6W	0.8398
N3—C11	1.426 (19)	O2W—H3W	0.8389
N3—C12	1.43 (2)	O2W—H4W	0.8393
C1—H1	0.9300	C9—O3	1.279 (16)
C2—C7	1.359 (18)	C9—O4	1.302 (14)
C2—C3	1.407 (17)		
			
N1^i^—Cd1—O10	96.6 (4)	C3—C4—C9	118.6 (11)
N1^i^—Cd1—O1	103.1 (4)	C5—C4—C9	119.8 (13)
O10—Cd1—O1	89.6 (3)	C4—C5—C6	119.5 (13)
N1^i^—Cd1—O3^ii^	90.6 (4)	C4—C5—C8	123.9 (12)
O10—Cd1—O3^ii^	172.6 (3)	C6—C5—C8	116.6 (12)
O1—Cd1—O3^ii^	86.9 (3)	C7—C6—C5	117.3 (12)
N1^i^—Cd1—O1W	169.1 (3)	C7—C6—H6	121.3
O10—Cd1—O1W	88.5 (3)	C5—C6—H6	121.3
O1—Cd1—O1W	86.5 (3)	C6—C7—C2	124.0 (13)
O3^ii^—Cd1—O1W	84.8 (3)	C6—C7—N2	132.0 (12)
N1^i^—Cd1—O4^iii^	85.9 (3)	C2—C7—N2	103.9 (12)
O10—Cd1—O4^iii^	93.7 (3)	O2—C8—O1	123.0 (14)
O1—Cd1—O4^iii^	170.0 (3)	O2—C8—C5	117.4 (13)
O3^ii^—Cd1—O4^iii^	88.7 (3)	O1—C8—C5	119.3 (11)
O1W—Cd1—O4^iii^	84.1 (3)	N3—C11—H11A	109.5
C8—O1—Cd1	130.4 (8)	N3—C11—H11B	109.5
H1W—O1W—H2W	112.2	H11A—C11—H11B	109.5
C10—O10—Cd1	127.5 (11)	N3—C11—H11C	109.5
C1—N1—C2	103.9 (12)	H11A—C11—H11C	109.5
C1—N1—Cd1^i^	118.8 (10)	H11B—C11—H11C	109.5
C2—N1—Cd1^i^	137.1 (9)	N3—C12—H12A	109.5
C1—N2—C7	106.8 (11)	N3—C12—H12B	109.5
C1—N2—H2	126.6	H12A—C12—H12B	109.5
C7—N2—H2	126.6	N3—C12—H12C	109.5
C10—N3—C11	123.2 (15)	H12A—C12—H12C	109.5
C10—N3—C12	119.1 (14)	H12B—C12—H12C	109.5
C11—N3—C12	117.5 (15)	O10—C10—N3	126.5 (16)
N1—C1—N2	113.5 (13)	O10—C10—H10	116.7
N1—C1—H1	123.2	N3—C10—H10	116.7
N2—C1—H1	123.2	H5W—O3W—H6W	105.9
C7—C2—N1	111.8 (12)	H3W—O2W—H4W	103.7
C7—C2—C3	120.0 (13)	O3—C9—O4	121.1 (12)
N1—C2—C3	128.2 (13)	O3—C9—C4	122.1 (12)
C4—C3—C2	117.7 (11)	O4—C9—C4	116.8 (13)
C4—C3—H3	121.2	C9—O3—Cd1^ii^	128.7 (8)
C2—C3—H3	121.2	C9—O4—Cd1^iv^	118.5 (7)
C3—C4—C5	121.6 (12)		

Symmetry codes: (i) −*x*, −*y*+1, −*z*+1; (ii) −*x*, −*y*, −*z*+1; (iii) *x*+1, *y*, *z*; (iv) *x*−1, *y*, *z*.

### Hydrogen-bond geometry (Å, °)

**Table:**

*D*—H···*A*	*D*—H	H···*A*	*D*···*A*	*D*—H···*A*
O1W—H1W···O2^iii^	0.84	1.92	2.757 (11)	177
O1W—H2W···O4^ii^	0.84	1.85	2.649 (12)	159
O2W—H3W···O1W	0.84	2.16	2.888 (9)	145
O2W—H4W···O1	0.84	2.00	2.811 (11)	162
O3W—H5W···O2^iii^	0.84	2.11	2.810 (12)	140
O3W—H6W···O2W^v^	0.84	2.29	2.766 (14)	117
N2—H2···O2W^vi^	0.86	2.18	2.970 (16)	152

Symmetry codes: (iii) *x*+1, *y*, *z*; (ii) −*x*, −*y*, −*z*+1; (v) −*x*+1, −*y*, −*z*+2; (vi) *x*, *y*+1, *z*.
